# Minimally invasive surgery - maximal exposure to research

**DOI:** 10.1186/1750-1164-5-3

**Published:** 2011-06-25

**Authors:** Ronald Matteotti, Seigo Kitano, Stanley Ashley

**Affiliations:** 1 Philadelphia, PA, USA; 2 Oita University, Yufu-city, Japan; 3 Brigham and Women's Hospital, Boston, MA, USA

## 

*Annals of Surgical Innovation and Research *enters its fourth year as an open access, fully on-line journal. In the last few years we witnessed dramatic changes in the world of science, clinically and from a research point of view. Social media and the ever growing world wide web became an integral part of science groups and discussion forums. These new technologies allow for a wide and fast dissemination of cutting edge research, clinical findings and are capable of incorporating audio-visual content unlike any other form of scientific publication, in our fast paced environment an important point not to underestimate. Its big advantage is the open access policy which allows everyone to see your research and dynamic manuscripts immediately upon acceptance. By incorporating an unlimited number of dynamic manuscripts we take the forefront in 'multimedia-publishing'. This new concept is especially useful in the field of minimally invasive surgery, robotic surgery and newer technologies, like NOTES (natural orifice transluminal endoscopic surgery), involving endoscopic devices where visual content is more important than a plain manuscript [1]. Scientists still fear to publish open access because of its costs and still not wide acceptance.

Why to pay for publication if I can publish for free in another journal?

These arguments were overthrown in the recent past where major grant institutions incorporate in their policies demands to publish preliminary results first online and open access. These developments really shaped the scientific world as we could witness in Northern Europe. The Norwegian research councils issued a statement that all research founded by public bodies *should *be published open access. The Swedish research council took this recommendation a step further and *mandated *to publish open access all research founded by grants issued after 2010 [2]. The NIH issued a statement in 2008 which clarifies their open access policy.

*SEC. 218. The Director of the National Institutes of Health shall require that all investigators funded by the NIH submit or have submitted for them to the National Library of Medicine's PubMed Central an electronic version of their final peer-reviewed manuscripts upon acceptance for publication, to be made publicly available no later than 12 months after the official date of publication: Provided, That the NIH shall implement the public access policy in a manner consistent with copyright law*[3].

Peer review is much faster and more efficient when compared to traditional publishing and we see an average of 30 days from receiving the manuscript to reach a first decision. This is especially important for time sensitive publications and research if you need results published for further grant applications. On the other hand peer review remains thorough by experts in the field and we see an approximate 35-40% acceptance rate. This highlights the high quality of research being published and in combination with the afore mentioned audio-visual component gives *ASIR *clearly an advantage over traditional 'paper-only' journals.

Costly subscriptions to individual journals with only a limited scope and 'subscriber access only' is frustrating and will soon be a thing from the past. Fee-based open access journals are on the rise and leading institutions like Harvard University, Massachusetts Institute of Technology, European Organization for Nuclear Research and many more incorporate policies and allow their faculty members access to these new publishing models. These trends are demonstrated by an increasing number of site visits, powered by search engines. At the beginning of 2011, over a period of four weeks, we saw 1855 visits from 98 countries with individual 3934 page views. The traffic sources can be broken down to 47.4% search engine based, 38% from referring sites and 14.6% direct traffic. Site visits were distributed between 98 countries and when broken down showed 562 visits in the United States of America with an increasing number from European countries, India and Asian territories.

The newly appointed Editors-in-Chief, together with a distinguished editorial board [4] are the foundation for attracting high quality submissions and guarantee peer review at the highest level. We are excited to step into a new decade together with our colleagues and being able to offer a cutting-edge forum for research and innovations across all specialties in the surgical field.

*Annals of Surgical Innovation and Research *is fully indexed by PubMed, PubMed Central, Scopus, Google Scholar and we aim to be indexed by Thompson Reuters (ISI) within the next few years.

We hope that clinicians, researchers and innovators will continue to consider *ASIR *as their primary source to publish and value the extraordinary capabilities this new concept offers - Incorporation of audiovisual content into your manuscript.
